# Detection of Metal Impurity Particles in Lead‐Acid Battery Electrolyte Based on Lens‐Free Digital Holography Technology

**DOI:** 10.1002/advs.202504991

**Published:** 2025-06-23

**Authors:** Liang Xue, Hao Zhou, Hao Chen, Zhihao Jiang, Chao Jiang, Haoyang Cui

**Affiliations:** ^1^ Shanghai University of Electric Power Shanghai 201306 China

**Keywords:** impurities detection, lead‐acid battery electrolyte, lens‐free digital holography(LDH), reconstruction distance, total gray‐scale difference(TGD)

## Abstract

Lead‐acid batteries, widely used in energy storage and power systems, are susceptible to performance degradation due to metal impurities such as iron, copper, and tin. Traditional methods like ICP‐OES are accurate but costly and complex. Therefore, this study proposes a new method based on lens‐free digital holography (LDH) to detect and distinguish metal impurities in lead‐acid battery electrolytes. The study uses a compact setup with a green LED light source and a CMOS camera to capture holographic images of impurities. The technique reconstructs clear particle images using angular spectrum algorithms and analyzes their reconstruction distances and gray‐scale differences for identification. Results show that copper particles have a reconstruction distance of 1406.4 µm and a total gray‐scale difference of 4.4681; tin particles have a reconstruction distance of 1414.85 µm and a total gray‐scale difference of 0.5344; iron hydroxide has a reconstruction distance of 1647.85 µm and the total gray‐scale difference of 13.5789. By combining these parameters, the method effectively distinguishes between copper, tin, and iron particles, even in mixed solutions. This cost‐effective and efficient method offers a promising alternative for rapid and accurate detection of metal impurities in lead‐acid battery electrolytes, with potential applications in battery maintenance and quality control.

## Graphical abstract

This study introduces a lens‐free digital holography (LDH) method for detecting metal impurities in lead‐acid battery electrolytes. Using a green LED and CMOS camera, the technique captures holograms and analyzes reconstruction distances and gray‐scale differences. It effectively differentiates copper, tin, and iron particles in lead‐acid battery electrolytes, offering a cost‐effective and efficient alternative to traditional methods like ICP‐OES.

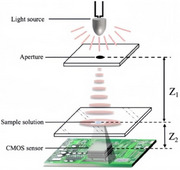

## 1 Introduction

Lead‐acid batteries, with a history exceeding 150 years, are widely utilized in various sectors such as energy storage,^[^ ^1^, ^2^, ^3^ ^]^ power systems,^[^ ^4^, ^5^, ^6^ ^]^ telecommunications,^[^ ^7^, ^8^, ^9^ ^]^ renewable energy,^[^ ^10^, ^11^, ^12^ ^]^ and military applications^[^ ^13^, ^14^, ^15^ ^]^ owing to their safety, reliability, high recyclability, and cost‐effectiveness. However, during operation, metal impurities such as iron, copper, and tin can accumulate in the electrolyte, significantly impacting battery performance.^[^ ^16^ ^]^ Iron contamination arises from the contamination of components during both production and usage, as well as from trace amounts of iron in the sulfuric acid used to prepare the electrolyte. When the iron concentration exceeds 0.01%, it causes damage to the battery plates, and when it surpasses 0.5%, severe self‐discharge occurs.^[^ ^17^ ^]^ Copper impurities are introduced through the corrosion of copper connectors during charge–discharge cycles, which can increase the negative electrode potential, reduce terminal voltage, and cause voltage imbalance in the battery pack when copper levels are elevated.^[^ ^18^ ^]^ Tin impurities, arising from the increased use of recycled lead in battery manufacturing or from the corrosion and wear of battery components, contribute to increased water consumption and deteriorated battery performance at higher concentrations.^[^ ^19^ ^]^ Given the detrimental effects of metal impurities on battery performance and lifespan, detecting these impurities in lead‐acid battery electrolytes has attracted significant attention in recent years.^[^ ^20^ ^]^ Various methods have been developed to detect metal particles in electrolytes, with notable differences in sensitivity, applicability, and operational complexity.^[^ ^21^ ^]^

Currently, metal impurity detection typically involves two main steps: first, the separation of metal impurities from the electrolyte,^[^ ^22^ ^]^ followed by their identification and quantitative analysis using advanced techniques. Several methods are available for quantitative analysis. Inductively coupled plasma optical emission spectrometry (ICP‐OES) provides high accuracy, precision, and a wide dynamic range.^[^ ^23^ ^]^ However, it is limited by the high cost of the spectrometer, its large size, and its complex operation. Additionally, spectral interferences from other elements in the sample may occur, requiring further correction methods.^[^ ^24^ ^]^ Atomic absorption spectroscopy (AAS) offers high accuracy but destroys the sample during analysis.^[^ ^25^ ^]^ Cyclic voltammetry (CV) also achieves high accuracy and precision but requires expensive equipment and sample pretreatment to eliminate interfering substances.^[^ ^26^ ^]^ In summary, there remains a lack of an economical, practical, and compact technology for detecting impurities in electrolytes. Although existing detection methods offer high sensitivity and accuracy,^[^ ^27^ ^]^ they often require costly equipment and skilled operators, which limits their widespread application across various scenarios. Therefore, the development of a cost‐effective, easy‐to‐operate technology capable of accurately detecting impurities in electrolytes is a promising area for future research. To this end, this paper proposes a novel method based on lens‐free digital holography (LDH) for detecting metal impurity particles in electrolytes.^[^ ^28^ ^]^ In the experiments, we utilized a compact, miniaturized lens‐free device that illuminates the electrolyte sample with an LED light source and records the data on a CMOS camera. By optically reconstructing the images, clear holographic images of the micro‐particles are obtained. Subsequently, by analyzing the reconstruction distance of the particle holograms and combining it with characteristic parameters of the holographic images,^[^ ^29^ ^]^ we can effectively distinguish and rapidly identify metal impurities in the electrolyte. Thus, lens‐free digital holography offers a novel approach for detecting and differentiating metal impurities in electrolytes, characterized by simplicity, rapid detection, high accuracy, and low cost. It holds significant potential for widespread use in practical liquid impurity detection.

## 2 Experimental Section

### 2.1 The Principle of Lens‐Free Digital Holography Imaging

**Figure 1:**
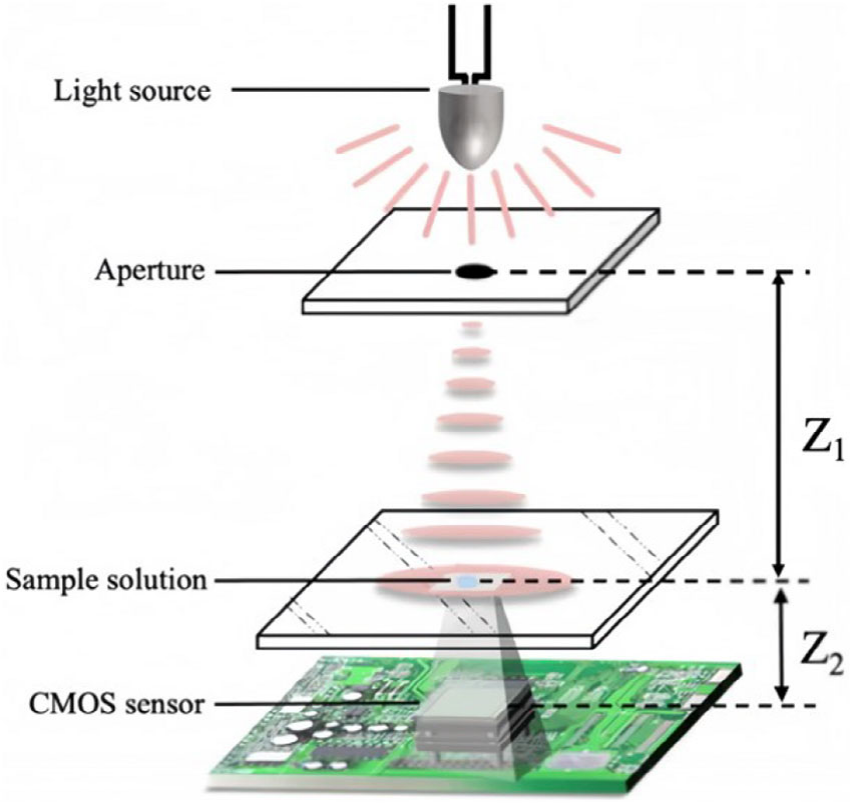
Lensless imaging schematic.

Lens‐free digital holography was an advanced imaging technique that eliminates the need for traditional optical lens systems to form images.^[^ ^30^ ^]^ The system consisted of a light source, an aperture, a sample, and a CMOS image sensor, as depicted in **Figure** **1**, which illustrates the setup of the lens‐free digital holography system. The imaging principle relied on the propagation of light as diverging spherical waves. A portion of the spherical wave was scattered by the sample, producing the object light, while the unscattered light serves as the reference. After propagating a distance (*Z*_2_), both the object and reference lights reach the CMOS image sensor (*Z* = *Z*_1_ + *Z*_2_), where they interfere and were recorded as a hologram. Here, *Z*_1_ represents the distance from the aperture to the sample, and *Z*_2_ represents the distance from the sample to the sensor. The system utilizes the CMOS image sensor to capture the interference pattern between the object and reference lights. This hologram contained all optical information of the sample, including both amplitude and phase information. In a lens‐free digital holography system, the distance from the light source to the sample should be significantly greater than the distance from the sample to the sensor (*Z*_1_ ≫ *Z*_2_), which expands the field of view and improves resolution. The light source used was a partially coherent LED, which reduces speckle noise typically generated by lasers, thereby enabling the capture of high‐quality holograms.

The mathematical derivation is outlined below:

The partially coherent light emitted by the LED source acts as the reference light.

(1) $$\begin{array}{c} R\left(x,y\right)=R_{0}\left(x,y\right)\exp\left[j\varphi_{R}\left(x,y\right)\right]\\ O\left(x,y\right)=O_{0}\left(x,y\right)\exp\left[j\varphi_{O}\left(x,y\right)\right] \end{array}$$

A fraction of this reference light was scattered by the sample under test, forming the object light.

In the equation,*R*_0_(*x*,*y*) and *O*_0_(*x*,*y*) denote the amplitude distributions of the reference and object light, respectively, while φ_*R*_(*x*,*y*) and φ_*O*_(*x*,*y*)represent their phase distributions. The reference and object light coherently combine to produce interference fringes, with the intensity *I*(*x*, *y*) given by:

(2) $$\begin{array}{c} I\left(x,y\right)=|R\left(x,y\right)+O\left(x,y\right){|}^{2}\;=I_{0}\left(x,y\right)+O\left(x,y\right)R^{*}\left(x,y\right)+O^{*}\left(x,y\right) \end{array}$$

**Figure 2:**
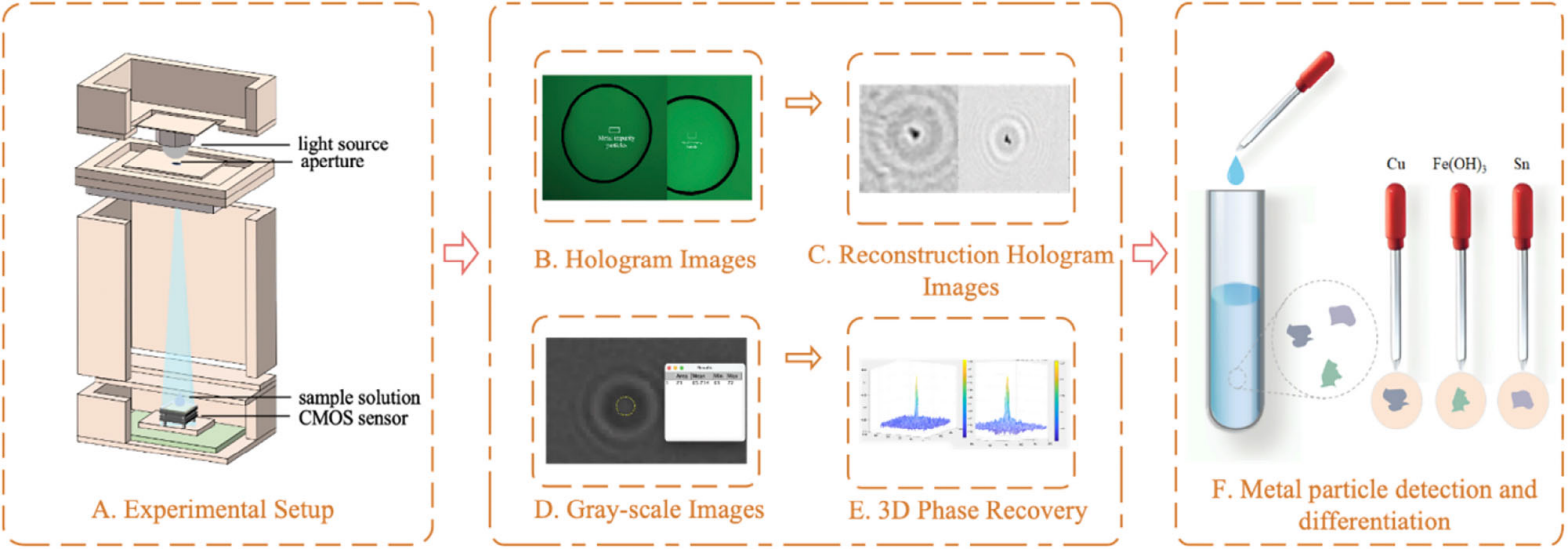
Experimental system. A) Experimental setup. B) Hologram Images. C) Reconstruction Hologram Images. D) Gray‐scale Images. E) 3D Phase Recovery. F) Metal particle detection and difference.

In the equation, R*(*x*, *y*) and O*(*x*, *y*) represent the conjugate of the reference light and the conjugate of the object light, respectively. The image information recorded by the CMOS image sensor can be calculated using the above formula, forming a series of holograms composed of alternating bright and dark circular fringes. For example, **Figure** **2**B shows the hologram of a sample solution containing copper particles. Additionally, in the model design, the scattering characteristics of the object should be considered. Based on the size, shape, and refractive index of the sample, as well as the wavelength of the incident light, the distribution and phase information of the scattered light were calculated using scattering theory.^[^ ^31^ ^]^ The corresponding specific formula is as follows:

(3) $$C_{sc}\left(\lambda \right)=\frac{2\pi }{k^{2}}\sum_{n=1}^{\infty }\left(2n+1\right)\left({\left|a_{n}\right|}^{2}+{\left|b_{n}\right|}^{2}\right)$$

here, *C_sc_* (λ)represents the scattering cross‐section, λ is the wavelength of the incident light, *k* is the wavenumber, and *a_n_* and *b_n_* are the Mie coefficients. These coefficients depend on the size, refractive index, and incidence angle of the particles. The Mie coefficients (*a_n_* and *b_n_*) describe the scattering and absorption behavior of the incident light wave at the particle surface. They can be obtained by solving the Mie equations. These coefficients contained information about the amplitude and phase of the electromagnetic field. In this study, the scattering equation for spherical particles was used to calculate the distribution and phase information of the scattered light, as shown below:

(4) $$\begin{array}{c} a_{n}=\frac{m_{rel}j_{n}\left(kr\right)-j_{n}\left(kr_{p}\right)}{m_{rel}h_{n}^{\left(1\right)}\left(kr\right)-h_{n}^{\left(1\right)}\left(kr_{p}\right)}\\ b_{n}=\frac{m_{rel}j_{n}\left(kr_{p}\right)-j_{n}^{\left(kr\right)}}{m_{rel}h_{n}^{\left(1\right)}\left(kr_{p}\right)-h_{n}^{\left(1\right)}\left(kr\right)} \end{array}$$

here, *m*_rel_ represents the relative refractive index, *j_n_* and *h_n_* ^(1)^ are the spherical Bessel functions, *r* is the particle radius, and *r_p_* is the wavelength parameter.

### 2.2 System Design

**Figure 3:**
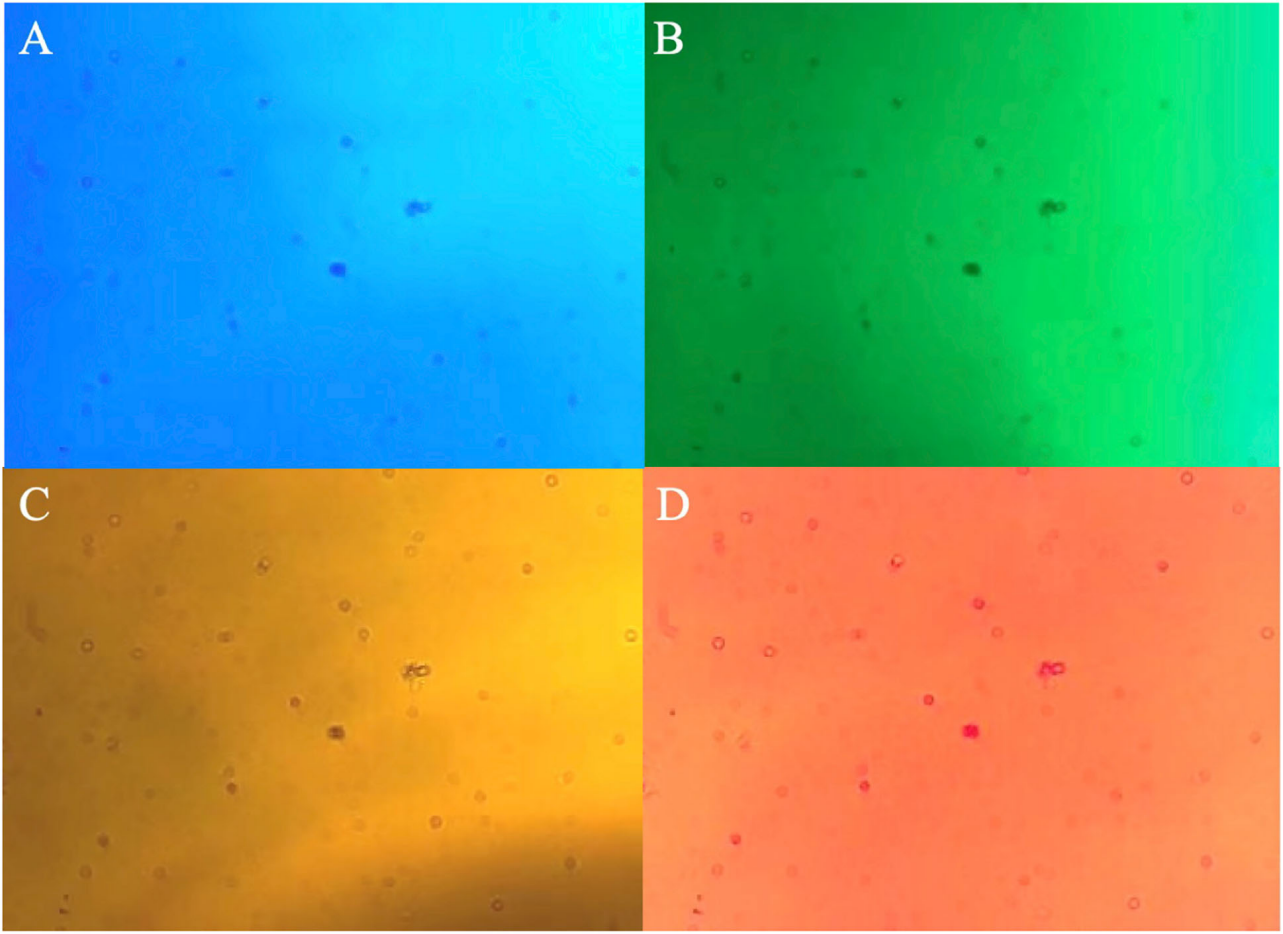
Holograms of the samples under different light sources. A) Blue light. B) Green light. C) Yellow light. D) Red light.

In this study, a lens‐free holographic imaging device, as shown in Figure 1, was utilized, with the experimental setup depicted in Figure 2A. The system primarily consisted of a green LED light source with a wavelength of 525 nm, an aperture, and a CMOS camera (model MV‐CB050‐11UC‐C), featuring a resolution of 2448 × 2048 pixels. Holograms were captured using blue light at 470 nm, green light at 525 nm, yellow light at 590 nm, and red light at 625 nm, as shown in **Figure** **3**. The selection of the 525 nm green light was due to its superior contrast and image clarity, which was essential for precise hologram capture and analysis.

The distance from the aperture to the sample was 60 mm, and the distance from the sample to the CMOS camera was 1 mm. During the experiment, a plastic pipette was first used to extract a sufficient amount of sample solution, which was then deposited onto a glass slide and inserted into the device. The sample was allowed to settle for a brief period. Subsequently, the emitting surface of the LED light source was positioned in close proximity to the 100 µm aperture. The light emitted from the source passed through the aperture and illuminated the sample below, while the hologram was captured by the CMOS camera and transmitted to a computer for further analysis. After reconstructing the hologram image, the result is shown in Figure 2C. Additionally, after gray‐scale processing of the hologram, a gray‐scale image was obtained, as illustrated in Figure 2D. As seen in Figure 2E, 3D phase reconstruction images of the particles were also acquired to observe the morphological distribution of the particles. Finally, through the analysis of the reconstructed and gray‐scale images, the metal impurity particles within the lead‐acid electrolyte could be effectively distinguished, as shown in Figure 2F.

### 2.3 Reconstruction Algorithm

Hologram reconstruction algorithms included the Fresnel transform method,^[^ ^32^ ^]^ convolution method,^[^ ^33^ ^]^ and angular spectrum method.^[^ ^34^ ^]^ The Fresnel transform method required only a single fast fourier transform (FFT), offering high computational efficiency. However, its application was limited by the Fresnel approximation, making it more suitable for short‐distance recording scenarios. The angular spectrum method involved two FFT operations and preserves high reconstruction quality over a wide range of propagation distances, making it versatile for various imaging applications. The convolution method required three FFTs and, while it excels in detail preservation at optimal reconstruction distances, it was primarily used for high‐precision imaging. In this study, the angular spectrum method was chosen for hologram reconstruction due to its moderate computational speed, precise description of diffraction phenomena in the frequency domain, and absence of approximations. The frequency‐domain transfer function of the wave is expressed as follows:

(5) $$\left\{\begin{array}{c} H_{z}\left(f_{x}f_{y}\right)=\left\{\begin{array}{c} T\sqrt{1-{\left(\frac{\lambda f_{x}}{n}\right)}^{2}-{\left(\frac{\lambda f_{y}}{n}\right)}^{2}}\cdot \sqrt{f_{x}^{2}+f_{y}^{2}}<\frac{n}{\lambda }\\ 0,\;\mathrm{other}\; \end{array}\right.\\ T=\exp\left[j\frac{2\pi n}{\lambda }Z\right] \end{array}\right.$$

here,*H_Z_* (*f_x_f_y_*) represents the optical transfer function in the frequency domain, n is the refractive index of the propagation medium, *f_x_* and *f_y_* are the spatial frequencies, *Z* is the propagation distance, and λ is the wavelength of the light source.

During the hologram reconstruction process, the reconstruction distance Z*_i_* refers to the distance between the object and the holographic plane, which directly affects the quality of the reconstructed image. Through the Fourier transform, different frequency components in the hologram correspond to different object depth information. Therefore, the reconstruction distance *Z_i_* must be set equal to the actual object distance *Z*_2_ to ensure that the reconstructed image was in focus.

### 2.4 Gray‐Scale Analysis

**Figure 4:**
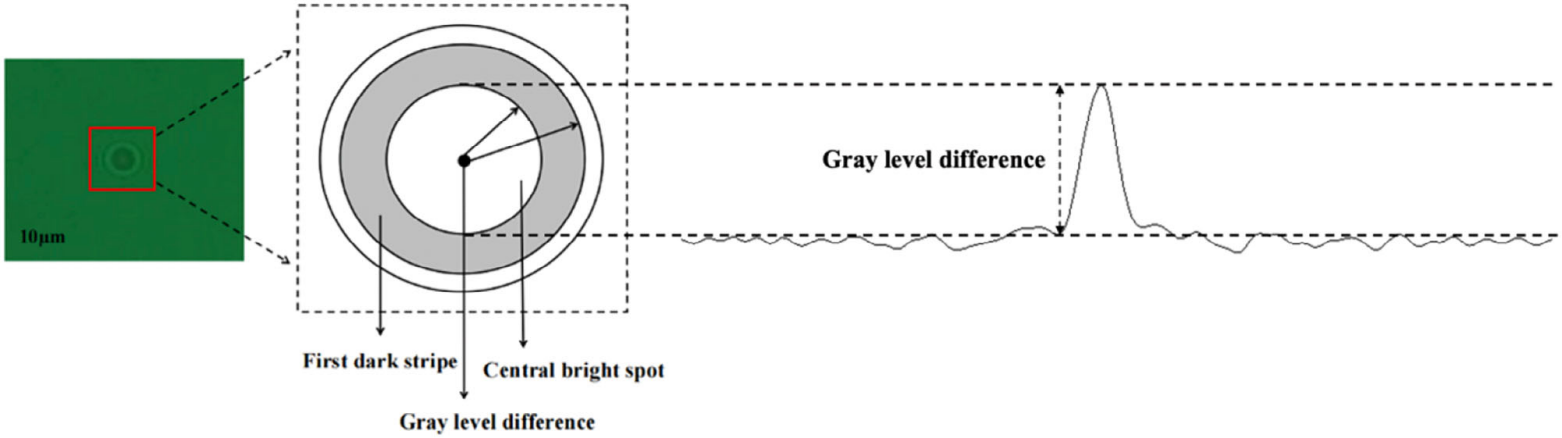
Gray‐scale image.

In a hologram, the total gray‐scale difference was defined as the average gray‐scale difference between the central bright spot and the first dark stripe of a particle,^[^ ^35^ ^]^ as illustrated in **Figure** **4**, which shows the total gray‐scale image. The gray‐scale value at a specific point on the hologram corresponds to the light intensity at that point. The gray‐scale was divided logarithmically, with a value of 0 representing black and 255 representing white. For particles of the same size, under illumination by a light source, a higher reflectivity leads to a significant increase in brightness at the central bright spot, while the brightness increase at the dark fringe was comparatively smaller. As a result, the brightness contrast between the central bright spot and the first dark stripe became more pronounced, thereby increasing the total gray‐scale difference. Consequently, by correlating the known reflectivity of the particles with their total gray‐scale values in the hologram, the particle type can be analyzed and inferred.

### 2.5 Preparation of Electrolyte Samples

The electrolyte samples used in this experiment were obtained from Yizhong Trading Co., Ltd. in Zhumadian, with an electrolyte mass percentage of 28%. Due to the acidic nature of the electrolyte, iron impurities were primarily present as iron ions. In contrast, copper and tin impurities do not react with sulfuric acid at room temperature and therefore exist as particulate matter. The radii of copper and tin particles were both 10 µm and can be observed using the lens‐free device. However, iron ions, with radii ranging from 0.064 to 0.074 nm, cannot be directly detected by this device. To address this, an adequate amount of sodium hydroxide solution (1 mol L^−1^ concentration) was added to the electrolyte samples to convert the iron ions into iron hydroxide precipitates. Since copper and tin particles do not react with sodium hydroxide at room temperature, the quantity of iron ions in the original solution can be determined by the amount of iron hydroxide precipitate formed. However, converting iron ions to iron hydroxide precipitates may introduce potential errors or interferences. To minimize these, the volume of sodium hydroxide solution added was carefully controlled to ensure complete conversion of iron ions into iron hydroxide while avoiding excess reagents. The reaction time was strictly regulated to allow the precipitation reaction to complete fully. The reaction temperature was maintained stable to prevent temperature – related interferences.

After conversion, the solution was settled for 60 s to facilitate iron hydroxide formation and sedimentation. The sample was then gently filtered to remove precipitated iron hydroxide, avoiding significant loss of the low – concentration precipitate. Deionized water use during rinsing was minimized to prevent sample solution dilution, and rinsing was done carefully to remove residual sodium hydroxide while retaining as much of the original solution as possible.

In this study, 0.01 g of copper, tin, and iron particles were each prepared into three separate sample solutions. The samples were then transferred to a glass slide using a graduated glass pipette. The distribution of particles in the electrolyte depends on their density: particles with a density lower than that of the electrolyte will float, while those with a higher density will sink. The samples were allowed to settle for 60 s before holograms were captured. These holograms were subsequently transmitted to a computer for reconstruction.

For each of the three solutions, ten particles were grouped together. The reconstruction distances of these particles were first recorded and averaged. The particles were then analyzed using ImageJ software to measure the total gray‐scale differences, which were also averaged. This process was repeated 20 times, and the final results were obtained by averaging the data from these twenty groups. It was important to note that all the data were obtained using our experimental setup.

## 3 Results

### 3.1 Distribution of Reconstruction Distances for Metal Impurity Particles in Lead‐Acid Electrolyte

**Figure 5:**
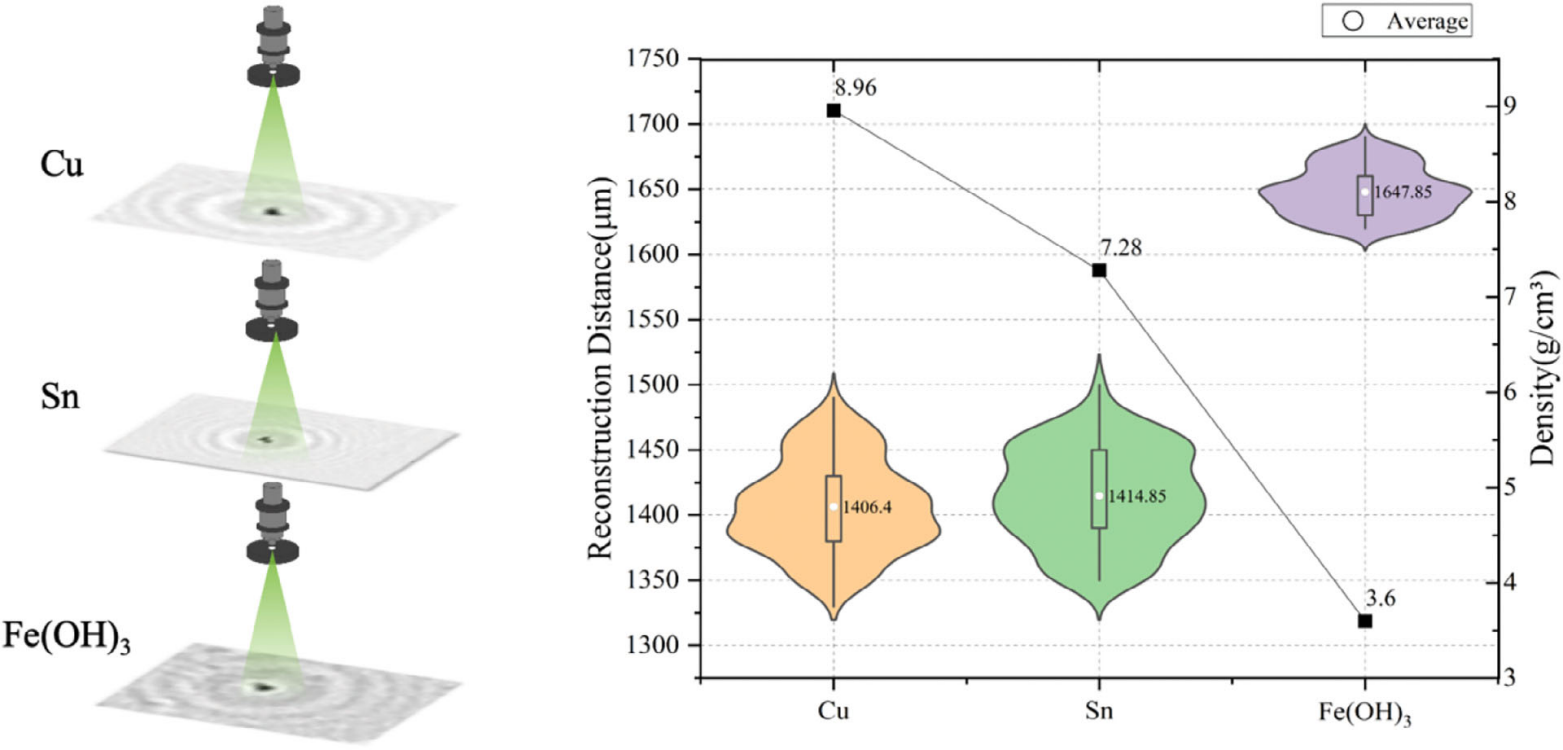
The distribution of reconstruction distances for different metal impurity particles in the electrolyte.

The distribution of reconstruction distances for different metal impurity particles in the electrolyte is presented in **Figure** **5**. Metal particles with a density greater than that of the electrolyte tend to sink, resulting in varying reconstruction distances based on their densities. Copper particles, with a density of 8.960 g cm^−^^3^, exhibit a reconstruction distance of 1406.4 µm, while tin particles, with a density of 7.28 g cm^−^^3^, show a reconstruction distance of 1414.85 µm. Iron hydroxide particles, with a density of 3.600 g cm^−^^3^, have a reconstruction distance of 1647.85 µm. It is evident that the reconstruction distances for copper and tin particles are quite similar, making it challenging to differentiate between them based solely on this parameter. In contrast, the reconstruction distance of iron hydroxide particles is markedly distinct from that of copper and tin, enabling effective separation of iron hydroxide particles from the others. Therefore, further analysis of gray‐scale values is essential for detecting and distinguishing these particles.

### 3.2 Gray‐Scale Distribution of Metal Impurity Particles in Lead‐Acid Electrolyte

**Figure 6:**
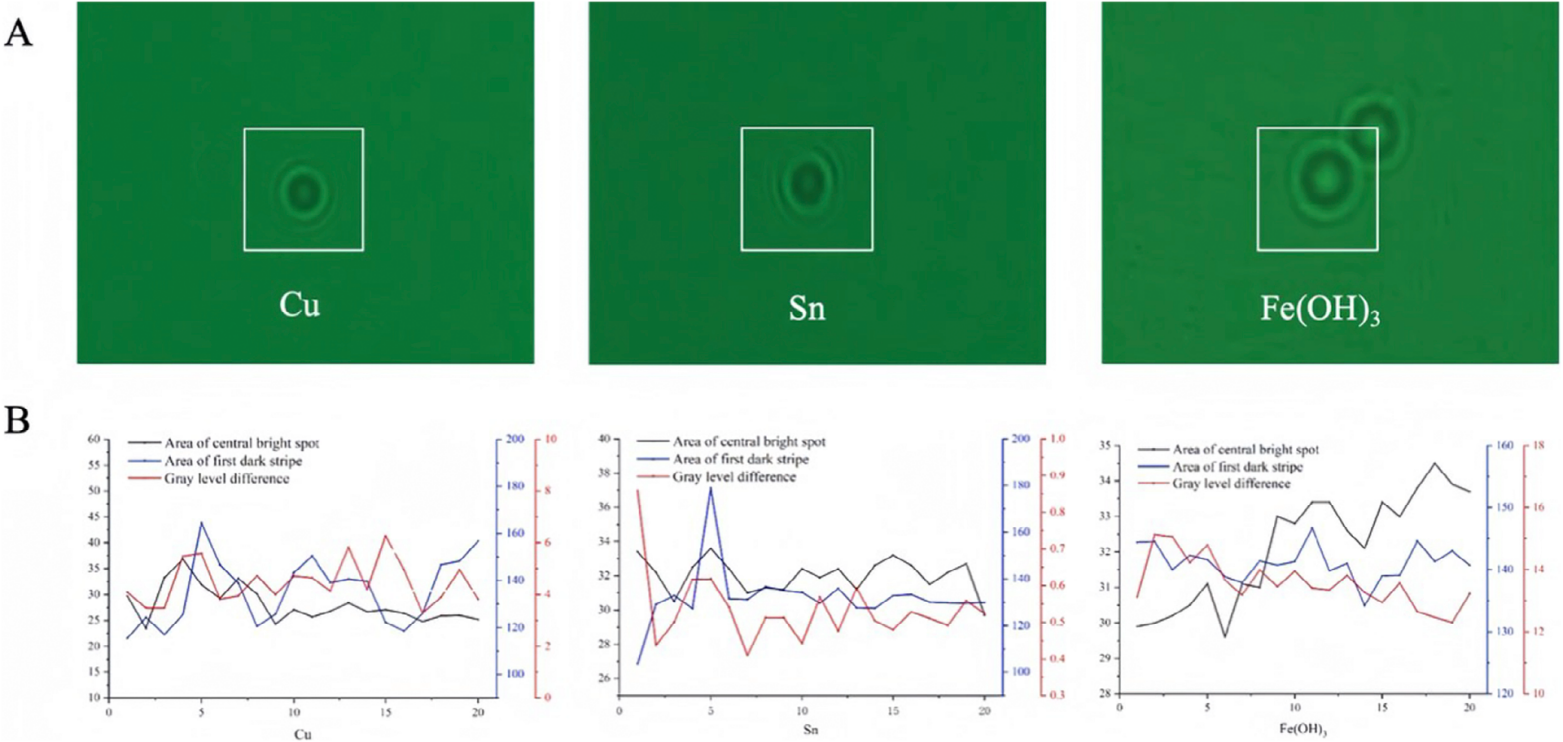
The distribution of reconstruction distances for different metal impurity particles in the electrolyte. A) Hologram images of Copper, Tin, and iron hydroxide Particles. B) The gray‐scale distribution of Copper, Tin, and iron hydroxide particles in the electrolyte.

The holographic images of copper, tin, and iron hydroxide particles in the electrolyte are presented in **Figure** **6**A. The gray‐scale distribution of these different metal impurity particles in the electrolyte is shown in Figure 6B. The total gray‐scale difference (TGD) of the particles can be calculated from the area of the central bright spot and the area of the first dark stripe in the figure. The results indicate that the average TGD values are 4.4681 for copper particles, 0.5344 for tin particles, and 13.5789 for iron hydroxide particles. The TGD for copper particles is primarily concentrated within the range of 2–8, for tin particles within 0.2–1, and for iron hydroxide particles within 12–15. Based on these findings, the combination of reconstruction distance and TGD as characteristic parameters allows for the differentiation of copper, tin, and iron particles in lead‐acid electrolytes.

Subsequently, in this study, 0.1 g of copper and 0.1 g of tin particles were added to 100 mL of lead‐acid electrolyte solution to investigate the distinction of impurities in a mixed sample containing both copper and tin particles.

### 3.3 Differentiation of Copper and Tin Impurity Particles in Mixed Sample Solutions

**Figure 7:**
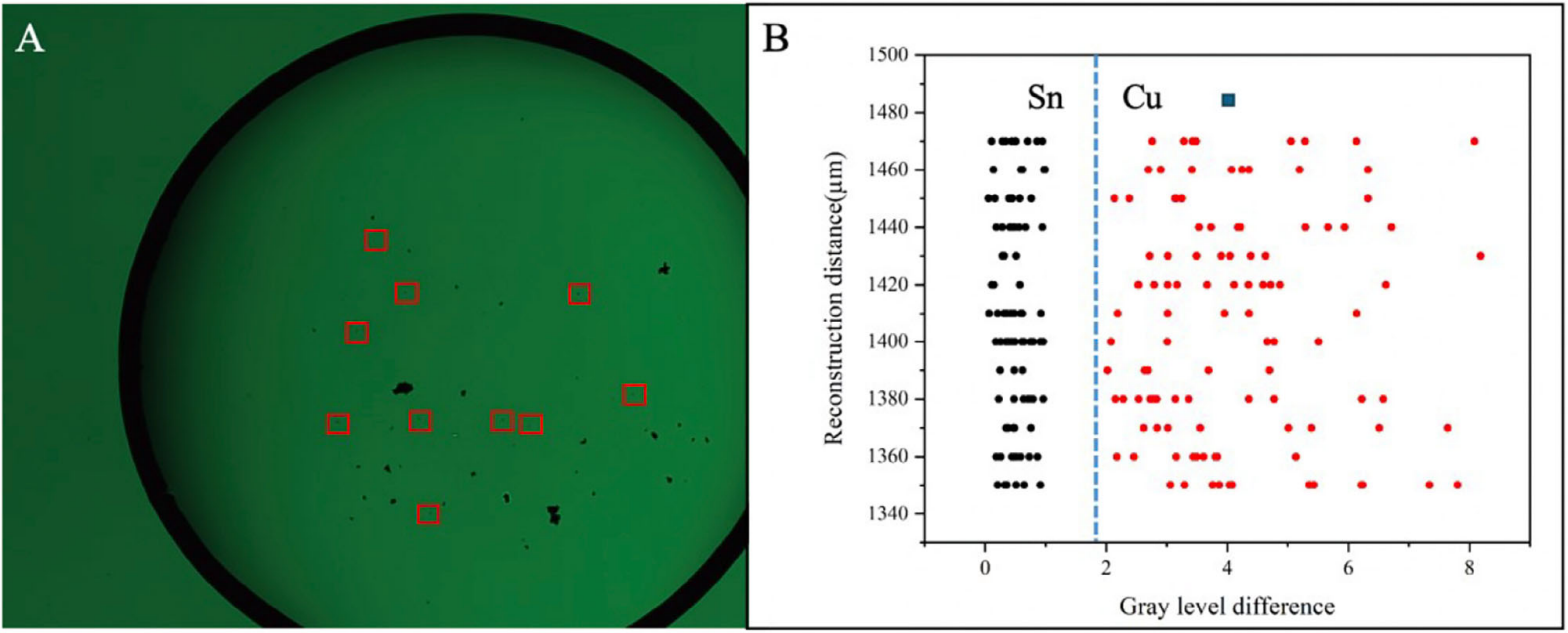
The reconstruction distances and total gray‐scale differences of 200 Particles were taken from the 20 groups of mixed solutions. A) Hologram image of mixed Solutions. B) Analysis of the reconstruction distances and total gray‐scale differences of 200 particles.

In the mixed sample solution, ten particles were selected from each group for measurement of the reconstruction distance and total gray‐scale difference, with experiments conducted across 20 groups. **Figure** **7** illustrates the reconstruction distances and total gray‐scale differences of 200 particles obtained from the 20 mixed solution groups. It is apparent that the reconstruction distances of these particles are closely grouped, ranging from 1350 to 1500 µm, which aligns with the characteristics of copper and tin particles. A subset of the particles exhibits a total gray‐scale difference ranging from 0.2 to 1, while another subset shows a total gray‐scale difference between 2 and 8. Based on these observations, it can be concluded that particles with a total gray‐scale difference below 1 are tin particles, and those with a total gray‐scale difference between 2 and 8 are copper particles. Thus, the differentiation of metal impurity particles in the electrolyte is achievable.

**Figure 8:**
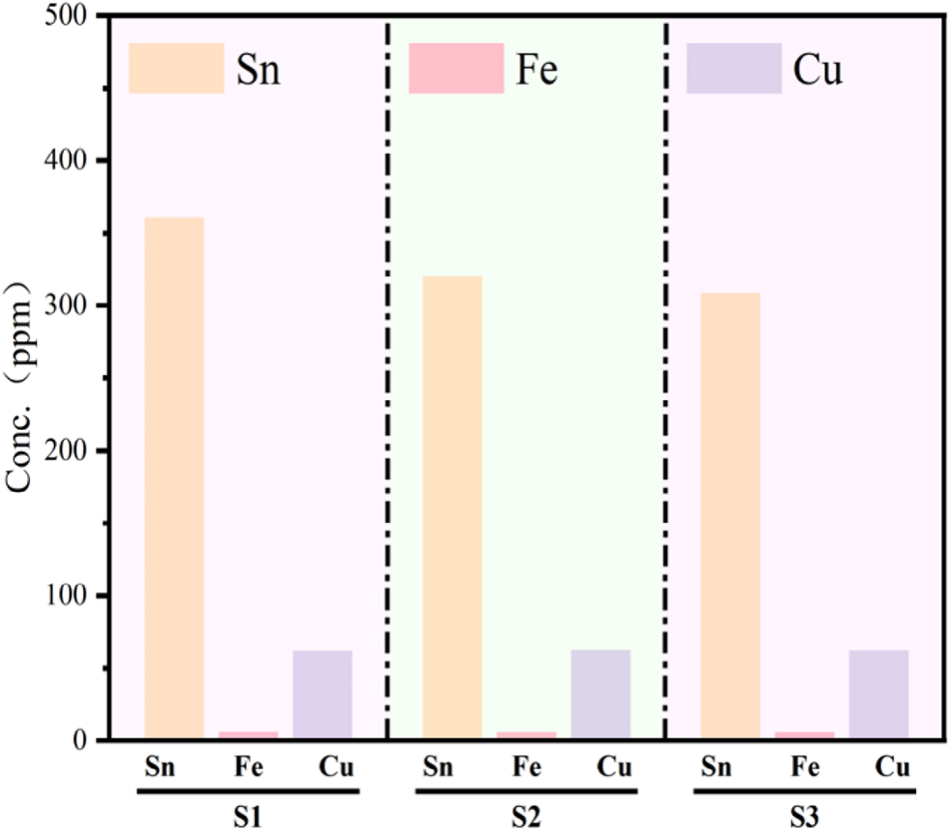
ICP‐OES analysis of mixed solutions.

To further validate the accuracy of the LDH method, ICP‐OES analysis was conducted on the mixed sample solution, as shown in **Figure** **8**. The results revealed a copper concentration of ≈62.1123 ppm and a tin concentration of ≈329.9397 ppm, with negligible concentrations of other metals. The LDH method successfully detected the copper and tin particles in the solution, and the results were consistent with those obtained through ICP‐OES. This demonstrates that the LDH method offers good accuracy in detecting metal impurities in lead‐acid battery electrolytes. However, the LDH method currently does not provide the same level of precise quantitative analysis as ICP‐OES. Future research could focus on optimizing the LDH method to enhance its quantitative capabilities, accuracy, and repeatability, thereby enabling a more comprehensive comparison with traditional methods such as ICP‐OES.

### 3.4 Morphological Distribution of Metal Impurity Particles in Lead‐Acid Electrolyte

**Figure 9:**
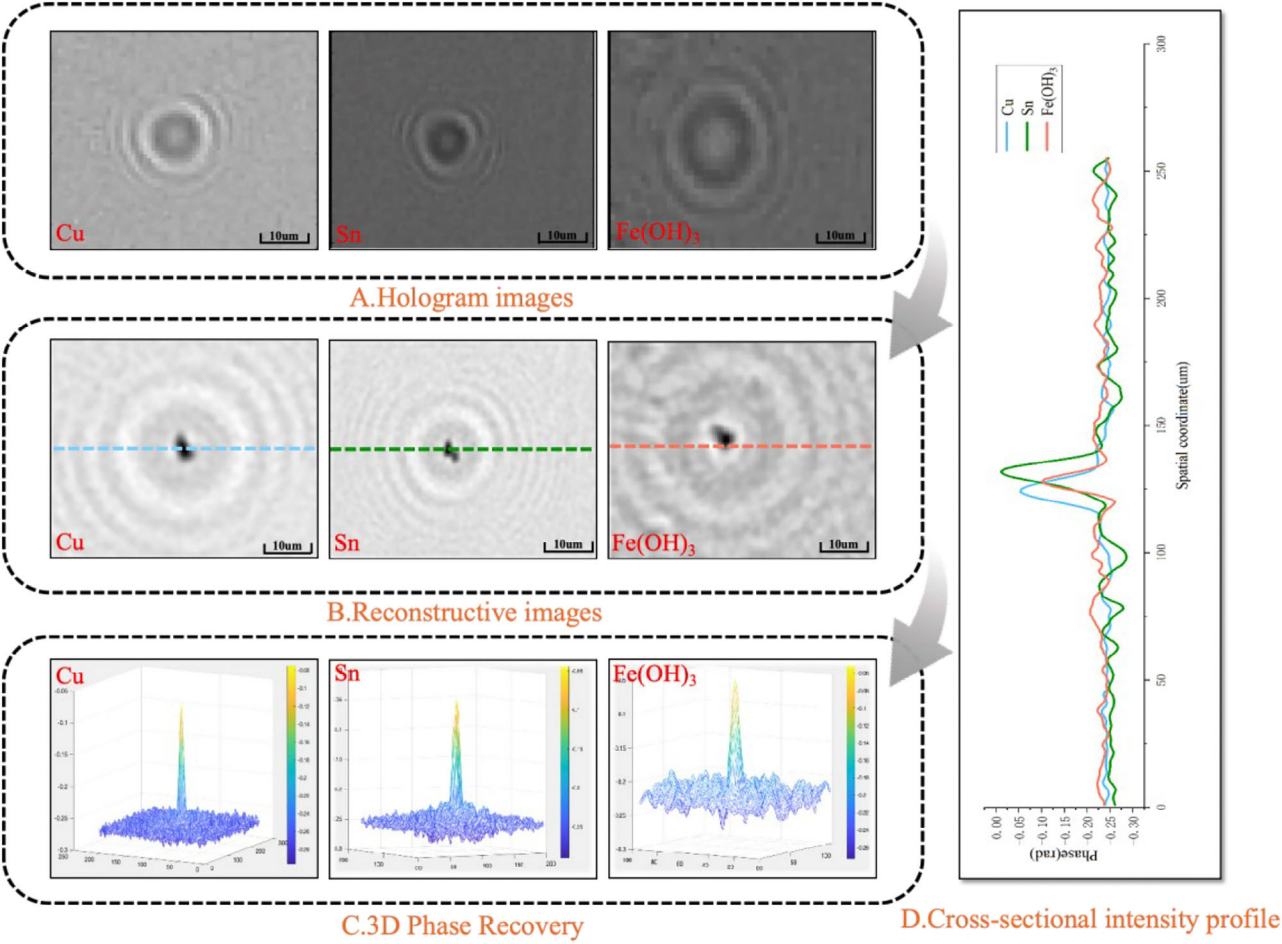
Hologram images and reconstructed images of metal impurity particles in the lead‐acid electrolyte. A) Holograms images. B) Reconstructed images. C) 3D Phase recovery. D) Cross‐sectional intensity profile along the dashed line direction.

Lens‐free digital holographic reconstruction provides information on the size, volume, and phase of individual particles. **Figure** **9** presents holograms and reconstructed images of metal impurity particles in lead‐acid electrolytes captured by the lens‐free holographic system, along with 3D phase maps and cross‐sectional intensity distribution profiles. The use of a partially coherent LED light source in this experiment, which enhances spatial coherence, results in holograms that exhibit richer textural details and distinct oscillatory patterns, encoding the phase information of the particles. Based on the 3D phase maps, the metal impurities in the lead‐acid electrolyte are observed to have both spherical and irregular shapes, with sizes predominantly ranging from 120 to 135 µm.

### 3.5 Analysis of Electrolyte Samples from Abandoned Lead‐Acid Batteries

**Figure 10:**
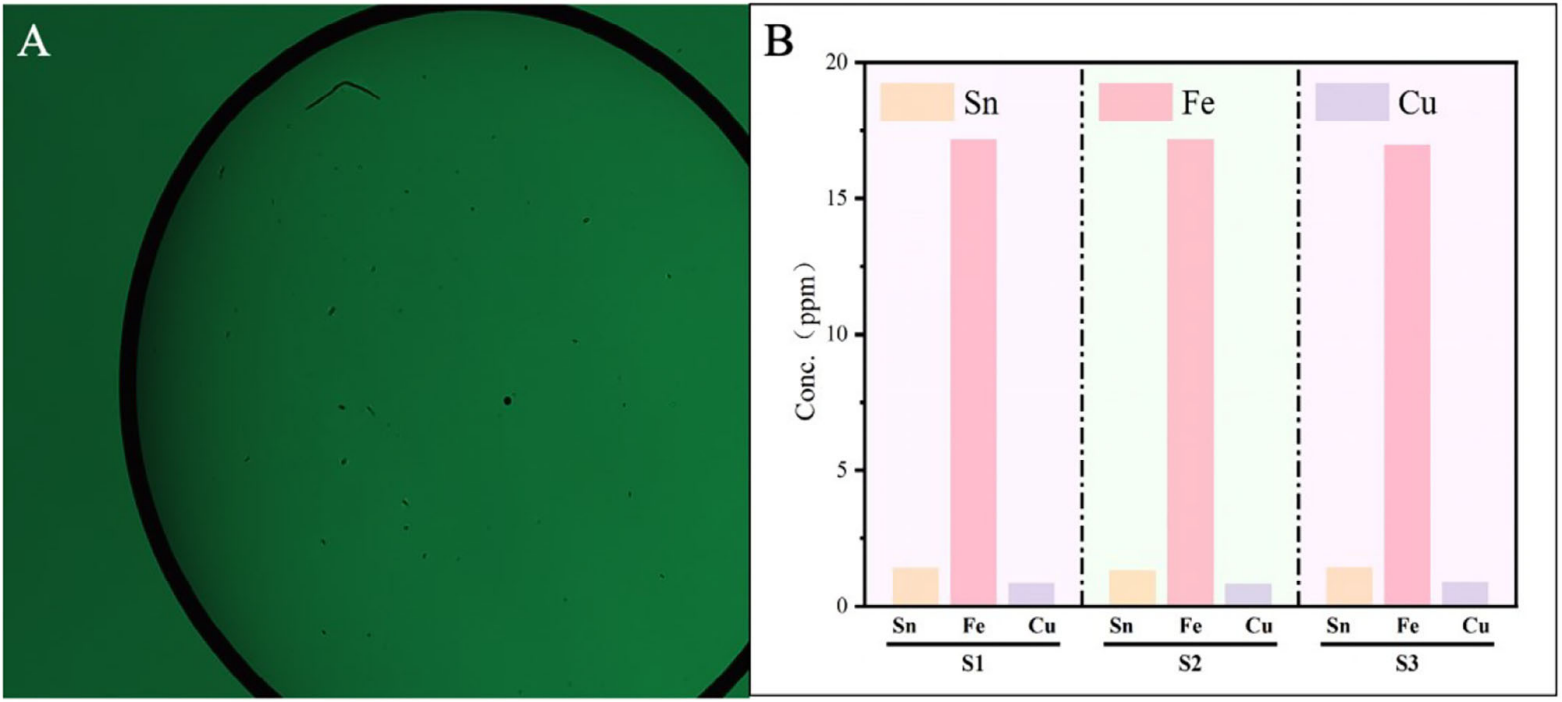
Analysis of electrolyte samples from abandoned lead‐acid batteries. A) Hologram image of the waste electrolyte samples. B) ICP‐OES analysis on the waste electrolyte samples.

To further validate the effectiveness and reliability of our LDH method for detecting metal impurity particles in lead‐acid battery electrolytes, we analyzed waste lead‐acid battery electrolyte samples, as shown in **Figure** **10**A. These samples were analyzed using ICP‐OES, with three separate analyses conducted to ensure the accuracy and reproducibility of the results. As shown in Figure 10B, the ICP‐OES analysis revealed copper concentrations of ≈0.8609 ppm, tin at 1.3951 ppm, and iron at 17.1219 ppm in the waste electrolyte.

**Figure 11:**
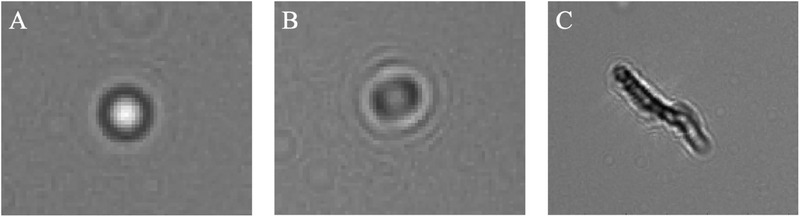
Interference factors in lead‐acid batteries electrolyte. A) Bubbles. B) The organic compounds. C) Cellulose particle.

Using the LDH method, a significant number of particles were identified, with reconstruction distances matching those of copper and tin. By applying the previously described gray value analysis method, these particles were accurately identified as copper and tin, respectively. However, some particles exhibited irregularly shaped long strips, which were inferred to be cellulose, as shown in **Figure** **11**A. Additionally, as shown in Figure 11B, there are some particles whose reconstruction distance exceeds 2000, and their shape is an irregular oval. These particles are attributed to the organic compounds in the waste liquid, as their density is much lower than that of the metal particles. Furthermore, particles with reconstruction distances ≈2500 exhibited highly regular concentric circles in the hologram, appearing either very dark or displaying a very bright central bright fringe, as shown in Figure 11C. These particles were inferred to be bubbles. The results obtained from the LDH method were in strong agreement with the concentrations measured by ICP‐OES, confirming that the LDH method effectively detects metal impurities at low concentrations. This indicates that the detection limit of the LDH method is comparable to that of ICP‐OES, typically reaching ≈1 ppm. The validation with real‐world samples further supports the reliability and applicability of our LDH method for detecting metal impurities in lead‐acid battery electrolyte.

## 4 Discussion

In this study, we performed a comprehensive analysis of potential metal impurity particles in lead‐acid electrolytes. To replicate the impurity conditions encountered in practical applications, copper, tin, and iron particles were manually introduced into pure electrolytes. Subsequently, a 1 mol L^−1^ sodium hydroxide solution was added to convert iron ions into iron hydroxide precipitates. This procedure allowed the preparation of sample solutions with consistent concentrations for detailed analysis.

Lens‐free holographic imaging analysis of the three sample solutions revealed that copper and tin particles, which have similar densities, both displayed reconstruction distances primarily within the range of 1350–1500 µm. In contrast, iron hydroxide particles exhibited a reconstruction distance centered ≈1650 µm. This variation in reconstruction distances provides an intuitive method for distinguishing copper/tin impurities from iron impurities in lead‐acid electrolytes. Specifically, copper and tin particles, being denser, tend to settle more quickly in the electrolyte, whereas iron hydroxide particles, with a lower density, sink more slowly, resulting in longer reconstruction distances in the holograms. This phenomenon indicates that reconstruction distance can serve as a key parameter for the preliminary differentiation of various metal impurity particles.

In addition to reconstruction distance, the total gray‐scale values of copper, tin, and iron hydroxide particles in the electrolyte were measured. The experimental results revealed that the total gray‐scale values for copper particles ranged from 2 to 8, for tin particles from 0.2 to 1, and for iron hydroxide particles from 12 to 15. These particles exhibited substantial differences in their gray‐scale values. These differences reflect their respective abilities to reflect and scatter light. Copper particles, with higher reflectivity, produced higher gray‐scale values in the holograms, while tin particles, with lower reflectivity, resulted in lower gray‐scale values. Iron hydroxide particles, due to their unique surface properties, showed the highest gray‐scale values. These gray‐scale differences provide another effective means of distinguishing between different metal impurity particles.

By combining reconstruction distance and gray‐scale value analysis, we were able to accurately detect and differentiate metal impurity particles in the electrolyte. In practical applications, metal impurities in lead‐acid battery electrolytes may be present in mixed forms, increasing the complexity of detection. However, using lens‐free holographic technology, we were able to swiftly identify and distinguish various metal impurity particles in mixed samples. For instance, in mixed samples, particles with a total gray‐scale value below 1 were identified as tin particles, those with values between 2 and 8 as copper particles, and those with values above 13 as iron hydroxide particles. This gray‐scale‐based differentiation method is not only straightforward and efficient but also highly accurate.

Furthermore, lens‐free holographic technology offers several significant advantages. First, the operation is simple, requiring no complex sample pretreatment; the sample can be directly placed onto a glass slide for detection. Second, the detection speed is fast, enabling the rapid acquisition and reconstruction of holograms. Finally, lens‐free holographic technology is cost‐effective, as essential components like LED light sources and CMOS cameras are inexpensive and widely available. These advantages make lens‐free holographic technology highly applicable, particularly in fields such as battery manufacturing, maintenance, and quality control.

## 5 Conclusion

This study presents a novel approach for detecting and differentiating metal impurity particles in lead‐acid battery electrolytes using lens‐free digital holography. The capability of this method to detect and distinguish metal impurities in the electrolyte was validated through a lens‐free digital holography setup. By analyzing the holographic images of various metal impurities, the reconstruction distances of three types of metal particles were measured, effectively differentiating iron hydroxide particles from copper and tin particles. Furthermore, through gray‐scale analysis of the copper and tin particles, which are difficult to distinguish, it was found that the total gray‐scale difference for tin particles is smaller than that for copper particles, and the total gray‐scale difference for copper particles is lower than that for iron hydroxide particles. These significant differences indicate that the gray‐scale‐based method for differentiating metal impurities in the electrolyte is not only simple and efficient but also highly accurate.

The findings of this study suggest that the LDH method can be effectively used to detect metal impurities in lead‐acid battery electrolytes, which is of great significance for battery maintenance. Timely identification of impurities such as copper, tin, and iron allows for prompt actions to minimize their detrimental effects. For instance, detecting iron impurities could lead to an investigation into contamination sources or internal component corrosion. Similarly, identifying copper or tin impurities could guide the replacement or purification of the electrolyte, thus preventing damage to the battery plates and prolonging the battery's lifespan.

Additionally, the LDH method offers valuable support for electrolyte management. By regularly monitoring impurity levels, it enables the identification of trends and the timely scheduling of maintenance activities. It also helps optimize battery performance by indicating when maintenance strategies need to be adjusted. For example, if sulfation is suspected due to impurities, the method can signal the need for more frequent equalization charging.

In conclusion, the LDH method provides an efficient means of detecting metal impurities in lead‐acid battery electrolytes, offering crucial technical support for improving battery performance and extending its lifespan. Future research will focus on further refining this technology, enhancing its accuracy in complex samples, and exploring its applications in fields such as environmental monitoring and material science. With its potential to become a low‐cost, multifunctional detection tool, lens‐free digital holography is expected to make a significant impact on both related research and practical applications.

## Conflict of Interest

The authors declare no conflict of interest.

## Author Contributions

Methodology was developed by L.X., H.Z., H.C., and Z.J. Supervision was provided by C.J. and H.C. All authors have read and agreed to the published version of the manuscript.

## Data Availability

The data that support the findings of this study are available from the corresponding author upon reasonable request.
